# Screening Co-Diagnostic Genes for Lung Adenocarcinoma and Myocardial Infarction and Analysis of the Molecular Functions and Drug Value of the Genes

**DOI:** 10.2174/0118715303374928250130113050

**Published:** 2025-02-11

**Authors:** Nannan Du, Mengting Liang, Zongjun Liu

**Affiliations:** 1 Department of Traditional Chinese Medicine, The Fourth People's Hospital of Bengbu, Bengbu, 233010, China;; 2 Department of Infection, Shanghai Longhua Hospital, Shanghai, 200032, China;; 3 Department of Cardiology, Shanghai Putuo District Central Hospital, Shanghai, 200062, China

**Keywords:** Lung adenocarcinoma, myocardial infarction, PPI network, molecular docking, MMP9, drug

## Abstract

**Background:**

Lung adenocarcinoma (LUAD) is the most common subtype of non-small cell lung cancer, and myocardial infarction (MI) is an acute cardiovascular disease resulting from the disruption of coronary blood supply. Recent studies have suggested that these two diseases may share common molecular mechanisms.

**Aims:**

The aim of this study was to discover common diagnostic genes for LUAD and MI and analyze their molecular functions and potential drug values by applying bioinformatics analysis.

**Objective:**

The objective was to provide a theoretical basis for further research on the pathological mechanisms of LUAD and MI, contributing to the development of novel diagnostic and therapeutic strategies for the two diseases.

**Methods:**

In this study, the datasets of LUAD and MI were obtained from TCGA and GEO databases, and differential expression analysis was performed to screen significantly differentially expressed genes (DEGs). Subsequently, disease-related genes were identified using WGCNA analysis, and the biological functions of these genes were explored by functional enrichment analysis. After screening key genes using the protein-protein interaction (PPI) network and the cytoHubba algorithm, biomarkers were determined by LASSO and SVM-RFE machine-learning methods. Finally, immune infiltration analysis and drug prediction were performed, and biomarker expression was verified by single-cell sequencing analysis.

**Results:**

A total of 158 differentially upregulated genes were identified between LUAD and MI. WGCNA analysis screened 86 genes that were significantly associated with both diseases and were enriched in an inflammatory response and immune regulation-related pathways, such as the IL-17 signaling pathway. Ten significant genes were identified by the PPI network and cytoHubba and then reduced to 4 using LASSO and SVM-RFE. Noticeably, MMP9 was significantly overexpressed in both diseases. Immune infiltration analysis showed that MMP9 was significantly related to multiple immune cell infiltration. Drug prediction and molecular docking analysis predicted Ilomastat and Osthole as the potential target drugs. Single-cell sequencing analysis revealed that MMP9 was high-expressed in the macrophages in LUAD tissues.

**Conclusion:**

This study identified MMP9 as a common diagnostic gene and potential therapeutic target for both LUAD and MI and revealed its role in inflammation and immune regulation through comprehensive bioinformatics analysis. These findings provided a theoretical basis for further research on the pathological mechanisms of LUAD and MI, contributing to the development of novel diagnostic and therapeutic strategies.

## 1 INTRODUCTION

Lung cancer is a major cause of cancer deaths, accounting for about 18% of total cancer deaths, and LUAD accounts for a relatively large proportion of lung cancer [1-3]. In recent years, there has been a gradual increase in the incidence of LUAD compared to other subtypes of lung cancer [4-6]. LUAD patients are prone to the development of metastatic foci, and most LUAD patients are already in the middle or late stage when diagnosed, which means that they have missed the optimal opportunity for surgical treatment [7, 8]. Clinically, chemotherapy with platinum-based drugs, such as cisplatin (DDP), is the main treatment for intermediate and advanced LUAD, as well as a variety of other cancers, either used alone or in combination [9, 10]. However, chemotherapy-induced toxicities are a great challenge that is difficult to be resolved during conventional chemotherapy, pointing to the need of developing novel therapeutic and prognostic markers.

Studies have found that complications of the cardiovascular system, including febrile myocarditis, arrhythmias, pericarditis, hypertension, thrombosis, and myocardial infarction (MI), may occur during LUAD treatment [11]. Similarly, cardiotoxicity caused by radiotherapy and chemotherapy drugs during lung cancer treatment may lead to complications of the cardiovascular system, such as myocardial injury and MI [12, 13]. Further studies showed that direct toxicity from radiotherapy and chemotherapy or indirect inflammatory response could all cause cardiovascular problems [14, 15]. Inflammatory markers C-reactive protein and interleukin-6 (IL-6) are significantly elevated in both LUAD and MI, and inflammatory factors may increase the risk of MI by promoting atherosclerosis and thrombosis [16]. Another study reported a significantly increased risk of venous thromboembolism (especially pulmonary embolism and deep vein thrombosis) that would further trigger MI among patients with LUAD [17]. These findings confirmed a potential link between LUAD and MI, but the specific molecular mechanisms have not been clearly revealed. Hence, mining diagnostic genes and analyzing their functions between LUAD and MI is urgent.

This study discovered the common diagnostic genes and examined molecular functions and drug values between LUAD and MI by bioinformatics analysis. We identified overlapping differentially expressed genes (DEGs) closely related to inflammatory response and immune regulation for the two diseases. In addition, drug prediction, molecular docking, and single-cell sequencing analysis revealed key diagnostic genes and their potential functions and drug values for both LUAD and MI, providing new understanding for further research on the association and treatment of these two diseases.

## 2 MATERIALS AND METHODS

### 2.1 Data Sources

The raw count with FPKM data of 474 cancer samples and 53 paracancer samples in the LUAD dataset was obtained from the University of California, Santa Cruz Xena (UCSC xena) database (https://xena.ucsc.edu/). The acute MI dataset GSE66360 from Gene Expression Omnibus (GEO, https://www.ncbi.nlm.nih.gov/geo/) was obtained to collect the circulating endothelial cell data of 49 patients with acute MI and 50 control cases. Two single-cell datasets of MI and LUAD were also obtained from the GEO database. Specifically, the LUAD single-cell dataset GSE117570 contained 3 tumor tissue samples, while the MI single-cell sequencing dataset GSE180678 contained 1 left ventricular myocardial tissue sample.

### 2.2 Differential Analysis

A total of 527 TCGA samples were assigned into tumor and control groups, and the gene expression differences between the two groups were calculated by the DESeq2 package to select significant DEGs under |log2FC| ≥ 1 and padj < 0.01 [18]. Ninety-nine GEO samples were assigned into tumor and control groups, and gene expression differences between the two groups were calculated by the Limma package to select DEGs under the criteria of |log2FC| ≥ 1 and padj < 0.01 [19, 20].

### 2.3 WGCNA Analysis

In order to better distinguish samples between tumor and control groups, a gene co-expression network was developed using the R package WGCNA [21]. In brief, the samples were divided into disease and control groups to ensure an accurate grouping of information in each sample. WGCNA analysis was performed under a soft threshold power, and the average connectivity of the network was close to 0.85. The minimum number of genes in each module was 200. Highly similar modules with a height threshold of 0.2 were merged. Finally, the modules showing the strongest positive correlation with the disease group were identified by performing correlation analysis on all the modules.

### 2.4 Functional Enrichment Analysis

The DEGs in the intersection between LUAD and MI groups were combined with the modular intersecting genes that showed the strongest positive correlation with two diseases in the WGCNA. Next, these DEGs were subjected to functional enrichment analysis for GO and KEGG by DAVID (https://david.ncifcrf.gov/) to analyze significantly enriched (*P* < 0.05) functions and pathways [22, 23].

### 2.5 Protein Interaction Network and Key Gene Screening

The intersecting genes were imported into the STRING database (https://cn.string-db.org/) to search for protein-protein interactions and construct a protein-protein interaction (PPI) network [24]. The obtained protein relationships were imported into Cytoscape (version 3.8.0) and analyzed by six algorithms of the cytoHubba plugin, namely, Maximal Clique Centrality (MCC), Maximum Neighborhood Component (MNC), Degree, Edge Percolated Component (EPC), Closeness, and Radiality. Genes screened by each algorithm that ranked the TOP 20 in terms of their importance in the PPI network were taken [25].

### 2.6 LASSO and SVM-RFE Screening Biomarkers

Initially, the expression data of the intersecting genes screened were extracted, and the variables were divided into disease and control groups. Using the glmnet package in R, LASSO regression analysis was performed on the 10 candidate genes and the model with the smallest error was selected as the final model [26]. Feature selection was performed using support vector machines (SVM). We used the e1071 package in R language to perform SVM-RFE analysis on the candidate genes, and the model with the smallest error was selected while eliminating unimportant feature genes by recursion. The genes selected by both LASSO and SVM-RFE models were taken.

### 2.7 Biomarkers and Immune Infiltration Correlation

The CIBERSORT package in R language was used to calculate the level of immune cell infiltration in the dataset samples based on the gene signature file LM22 from the CIBERSORT official website (https://cibersortx.stanford.edu/). Rank-sum test was used to reveal significant differences in immune infiltration between the tumor and control groups. The correlation between biomarkers and immune cell infiltration status was calculated based on the Spearman correlation coefficient [27].

### 2.8 Targeted Drug Prediction and Molecular Docking

The gene sets were enriched and analyzed by Enrichr R package (https://cran.r-project.org/web/packages/enrichR/index.html), and biomarker-targeted drugs were predicted through the Drug Signatures Database (DSigDB, http://dsigdb.tanlab.org/DSigDBv1.0/). Crystal structures of receptor proteins were obtained from the Uniprot database (https://www.uniprot.org/). Based on the drug prediction results, the 3D structures of the targeted drugs were downloaded from the PubChem website (https://pubchem.ncbi.nlm.nih.gov/) as ligands. We used PyMOL software to dehydrate, hydrogenate, and remove small molecules for all molecules [28]. The 3D structure energy of the targeted drug was minimized by ChemBioOffice software, followed by receptor protein-drug molecule docking and screening using AutoDocktools under binding energy < -5 kcal/mol and hydrogen bond length < 3.5Å.

### 2.9 Biomarker Expression in Single-Cell Sequencing

The Seurat 3.2.3 package was utilized to read the scRNA-seq data and identify cells with gene numbers between 200 and 4000 and with a proportion of mitochondrial genes greater than 10% [29, 30]. After data normalization with the SCTransform method, PCA downscaling was performed in the harmony package to remove batch effects between different samples [31]. After UMAP dimensionality reduction, we used the FindNeighbors function to construct a KNN graph based on Euclidean distance and the first 30 principal components and used FindAllMarkers to identify specific high-expressed genes among different subgroups under logfc.threshold = 0.2, min.pct = 0.1,only.pos = T. Finally, the cells were clustered by the FindCluster function into subpopulations at a resolution of 0.1. Cell types were determined based on the expressions of the marker genes in the CellMarker database (http://biocc.hrbmu.edu.cn/CellMarker/).

## 3 RESULTS

### 3.1 A Total of 158 Genes were Differentially Upregulated in LUAD and MI

**Fig. (1):**
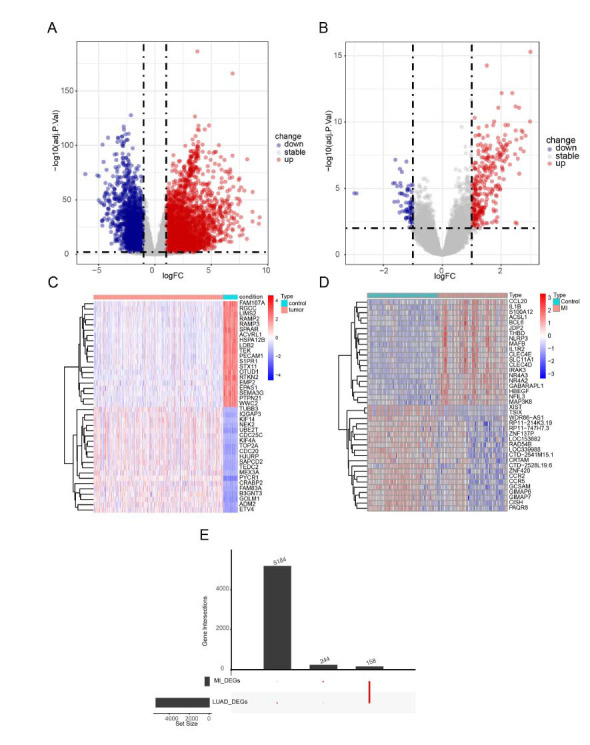
158 genes were differentially upregulated in both diseases. **A**: Volcano plot of differential expression of LUAD genes, horizontal coordinates represent differential expression fold log2FC, vertical coordinates represent -log10 (adj.P.Val); each point in the plot represents a gene, and blue color in the left half of the plot is for down-regulated genes, and red color in the right half of the plot is for up-regulated genes. **B**: Heat map of LUAD gene expression, blue color represents low expression, red color represents high expression. **C**: Volcano plot of MI gene differential expression. **D**: Heat map of MI gene expression. **E**: The upset plot, the left bar is the number of each subset of genes, and the top bar is the number of each intersection of genes.

We screened 5342 DEGs (3273 upregulated genes and 2069 downregulated genes) in LUAD (Fig. **1A**). The top 20 significantly upregulated and downregulated genes (40 genes in total) were visualized in the expression heatmap (Fig. **1B**). A total of 402 DEGs (317 upregulated genes and 85 downregulated genes) in MI were discovered (Fig. **1C**). The top 20 significantly upregulated and downregulated genes (40 genes in total) were visualized in the expression heatmap (Fig. **1D**). There were 158 overlapping DEGs between the two diseases (Fig. **1E**).

### 3.2 WGCNA Screening for Disease-Related Genes

**Fig. (2):**
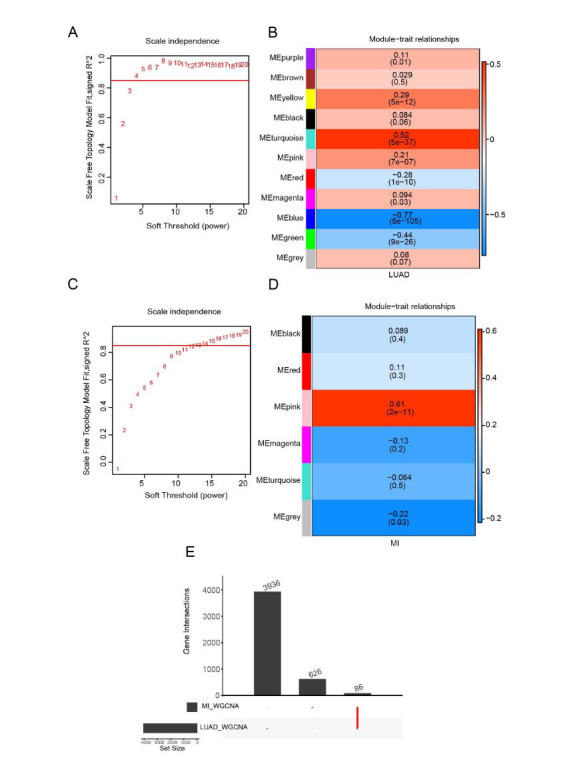
WGCNA screening for disease-related genes. **A**: LUAD dataset soft threshold screening plot, screening the minimum value of R^2 above 0.85 as the soft threshold for constructing the topological network. **B**: Module-trait correlation plot of LUAD dataset, where the horizontal coordinate is the trait, the vertical coordinate is the module, the numbers in the squares are uncorrelation coefficients, and the numbers in the parentheses are the significance *P*-values, with the red color representing the positive correlation and the blue color representing the negative correlation. **C**: The soft-threshold screening plot for the MI dataset. **D**: Module-trait correlation plot for MI dataset. **E**: The upset plot of genes in LUAD and MI.

We performed WGCNA analysis on LUAD and MI data, respectively, under an intercept height of 0.85 to set the minimum number of module genes as 200. Under the optimal soft threshold of 4 (Fig. **2A**), 11 modules were obtained for the LUAD dataset, and the module with the strongest positive correlation was MEturquoise (R = 0.52, *P* = 5e -37) (Fig. **2B**). The optimal soft threshold for the MI dataset was 13 (Fig. **2C**), under which 6 modules were sectioned, and MEpink showed the strongest positive correlation with MI (R = 0.61, *P* = 2e-11) (Fig. **2D**). The genes in the intersection of the two modules were taken (Fig. **2E**).

### 3.3 Functional Enrichment Analysis of the Candidate Genes

**Fig. (3):**
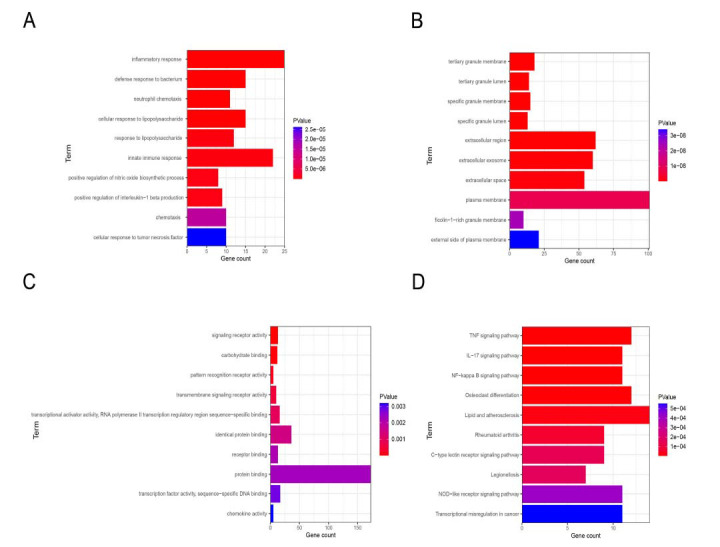
Functional enrichment analysis of candidate genes. **A**: The bar graph of gene GO enrichment analysis BP. **B**: The bar graph of gene GO enrichment analysis CC. **C**: The bar graph of gene GO enrichment analysis MF. **D**: The bar graph of gene KEGG enrichment analysis, with the horizontal coordinates representing the number of genes contained within the entries and the colors representing the significance *P*-values, which increase in significance from blue to red.

DEGs and WGCNA results were combined to collect 232 common genes. Functional enrichment analysis of these genes showed that inflammatory response, neutrophil chemotaxis, tertiary granule membrane, specific granule membrane, and IL-17 signaling pathway were significantly enriched, and that most of these entries were associated with immunity, inflammation, tumor, and cardiovascular diseases (Figs. **3A**-**D**).

### 3.4 Protein Interaction Network and Screening for Important Genes

**Fig. (4):**
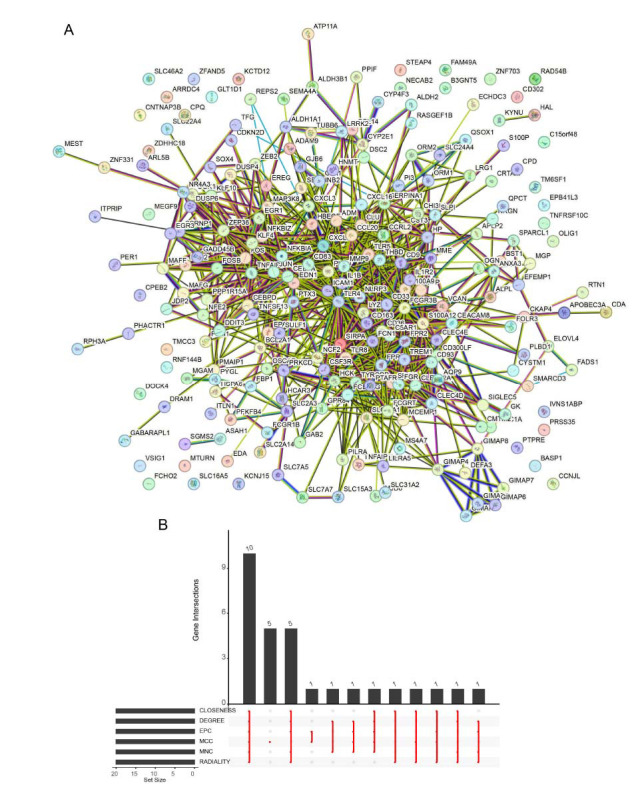
Protein Interaction Network and Important Genes Screening. **A**: PPI network graph of 232 intersecting genes; each circle in the graph represents a protein corresponding to a gene. **B**: intersection upset graph of the TOP 20 genes in terms of importance obtained by 6 algorithms of cytoHubba.

We constructed the PPI regulatory network by STRING database using the 232 common genes (Fig. **4A**). The obtained protein regulatory relationships were loaded into Cytoscape software, and 10 intersecting genes were taken from the intersection of the TOP 20 important genes identified by the six algorithms using the cytoHubba plug-in (Fig. **4B**).

### 3.5 Two Machine Learning Algorithms for Mining Tumor Signature Genes

**Fig. (5):**
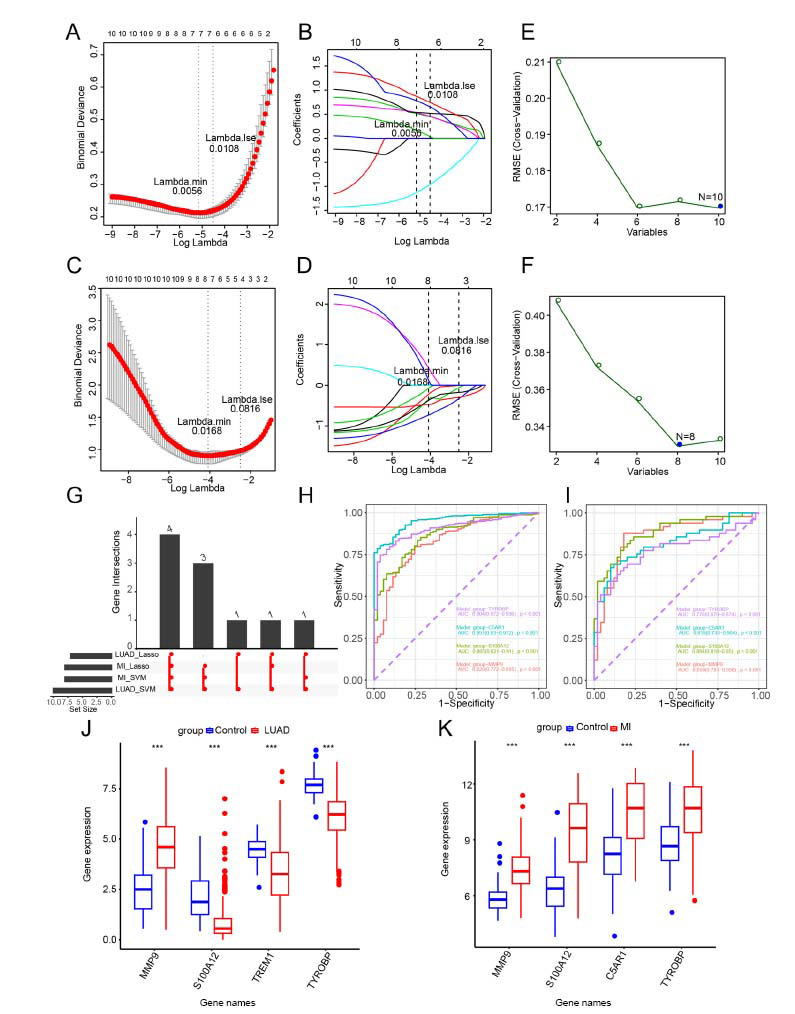
Two machine learning screening for tumor signature genes. **A**: Plot of Lasso penalty term parameters for the LUAD dataset, with log(lambda) values in horizontal coordinates and degrees of freedom in vertical coordinates, representing the cross-validation error and the position where the cross-validation error is expected to be minimized in the actual analysis. in the plot, the dashed position on the left side is the position with the smallest cross-validation error, and based on this position (lambda.min), the topmost cross-validated cross-coordinate is determined, log (Lambda), and the number of characterized genes is shown on the top. **B**: Plot of Lasso regression coefficients for the LUAD dataset, with log(lambda) in the horizontal coordinate and the coefficients of the genes in the vertical coordinate, showing how the coefficients of the different variables change with λ-penalty. **C**: Plot of Lasso penalty term parameters for the MI dataset. **D**: Plot of Lasso regression coefficients for the MI dataset. **E**: Plot of SVM-RFE generalized error *versus* the number of features for the LUAD dataset, with the horizontal coordinate being the number of genes included within the model at each iteration, and the vertical coordinate being the root-mean-square error, which represents the model optimality at the smallest value. **F**: Plot of SVM-RFE generalized error *versus* the number of features for the MI dataset. **G**: Lasso *vs.* SVM-RFE to get the intersection of feature genes upsets plot. **H** and **I**: LUAD *vs.* MI dataset ROC curves for 4 feature genes. **J** and **K**: Box line plots of expression of 4 feature genes in LUAD *vs.* MI dataset. ***p > 0.001.

LASSO regression analysis was performed on the 10 candidate genes. A 7-gene model showing the smallest error (lambda.min = 0.0056) was selected as the final model for the LUAD dataset (Figs. **5A** and **B**). Similarly, an 8-gene model showing the smallest error (lambda.min = 0.0168) was determined as the final model for the MI dataset (Figs. **5C** and **D**). The SVM-RFE analysis developed a model containing feature genes with the smallest model error for the LUAD dataset when N = 10 (Fig. **5E**). The model error was the smallest when N=8 for the MI dataset, therefore an 8-gene model was developed (Fig. **5F**). There were a total of 4 intersecting genes in the above four models (Fig. **5G**). The ROC analysis of the 4 genes in the two datasets showed that they all had a strong predictive ability in the two diseases (Figs. **5H** and **I**). The box line plot showed that only the MMP9 gene was significantly high-expressed in both datasets (Figs. **5J** and **K**).

### 3.6 Immune Infiltration and MMP9 Correlation

**Fig. (6):**
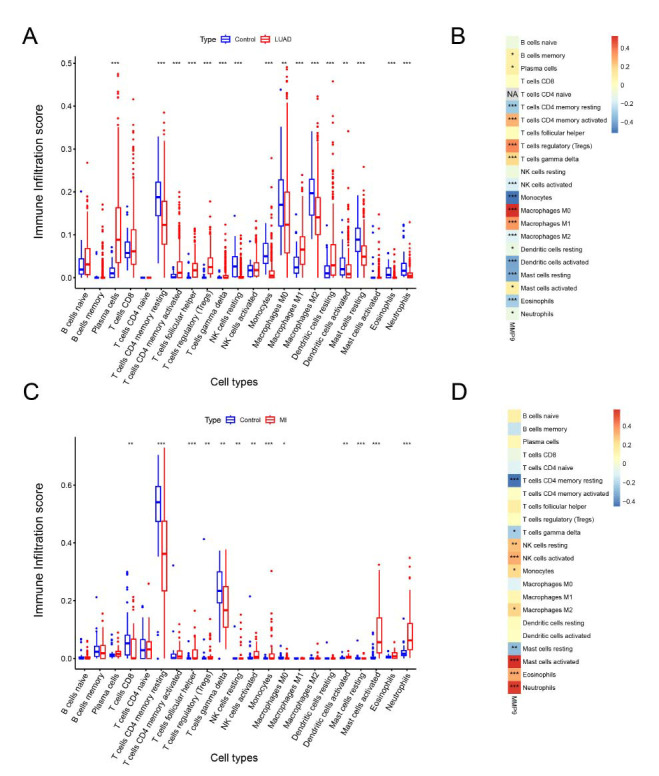
Immune infiltration and MMP9 correlation. **A**: Box line plot of immune cell infiltration in 22 of the LUAD dataset CIBERSORT. **B**: Spearman correlation analysis of biomarkers with immune infiltration. **C**: Box line plot of immune cell infiltration in 22 of the MI dataset CIBERSORT. **D**: The spearman correlation analysis of biomarkers with the immune infiltration situation. *p > 0.05, **p > 0.01, ***p > 0.001

CIBERSORT analysis results showed that the infiltration of 16 immune cells was significantly different between both tumor and control groups in the LUAD dataset (Fig. **6A**). The calculation of correlation coefficients between the expressions of the biomarkers and immune cell infiltration scores revealed that 17 immune cell infiltration profiles were significantly correlated with MMP9 expression (Fig. **6B**). Immune infiltration analysis on the MI dataset demonstrated that the infiltration of 13 immune cells was significantly different between both disease and control groups (Fig. **6C**). After calculating the correlation coefficients between biomarker expressions and immune cell infiltration scores, it was found that 10 immune cell infiltration profiles were significantly correlated with MMP9 expression (Fig. **6D**).

### 3.7 Drug Prediction and Molecular Docking

**Fig. (7):**
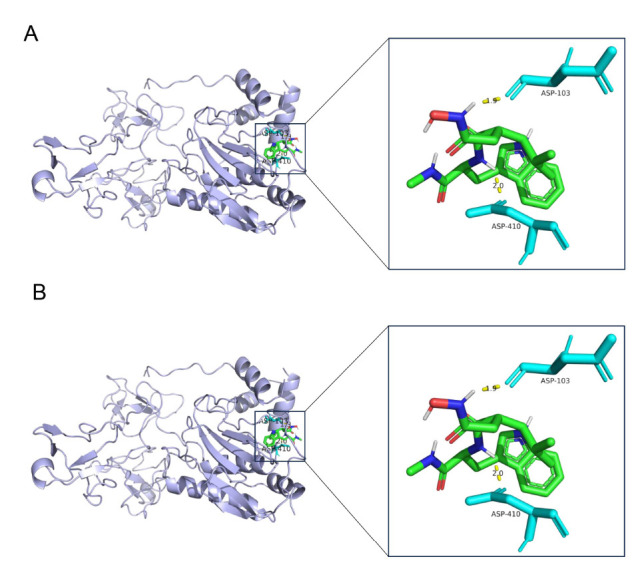
Drug prediction and molecular docking. **A**: MMP9 docking results with Ilomastat molecule. **B**: MMP9 and Osthole molecule docking results. In the figure, blue molecules are receptor proteins, green molecules are small drug molecules, and lime green are amino acids. The binding hydrogen bond between the receptor protein and the drug small molecule is indicated by the yellow dotted line, and the upper number is the hydrogen bond length in angstroms (Å).

The biomarker-targeted drugs were predicted in the DSigDB database. After screening, Ilomastat (an inhibitor of the MMP family) and Osthole (for the treatment of cardiovascular diseases) were finally chosen as the drug molecules. A close binding relationship between Osthole-MMP9 and Ilomastat with an absolute binding free energy higher than 5 kcal/mol was clearly observed, suggesting a stable binding between the two (Figs. **7A** and **B**, Table **1**).

### 3.8 Single-Cell Mapping of LUAD Tissue

**Fig. (8):**
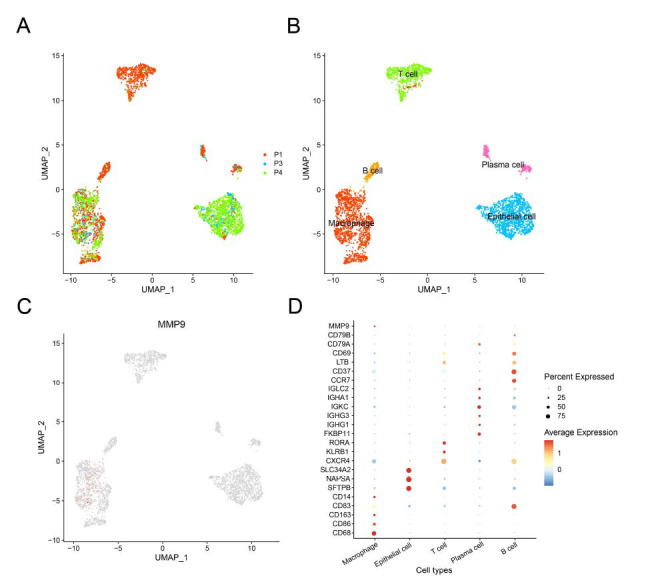
Single-cell mapping of lung adenocarcinoma tissue. **A**: Distribution of different samples after de-batching. **B**: UMAP visualization of the distribution of different cell types. **C**: Distribution of MMP9 gene in cells. **D**: MMP9 as well as different cellular marker gene expression levels.

The LUAD single-cell sequencing data were used to cluster 5 cell clusters after cell filtering, normalization, de-batching, and dimensionality reduction (Fig. **8A**). Annotation of these cell clusters based on the expressions of classical marker genes determined five types of cells, including macrophages, epithelial cells, TT cells, plasma cells, and B cells (Fig. **8B**). By plotting the distribution of MMP9 cells, it was found that MMP9 cells were high-expressed, mainly in macrophages (Fig. **8C**). Subsequent bubble plots based on cell markers and MMP9 expressions were plotted to reconfirm the gene expression profile (Fig. **8D**).

## 4 DISCUSSION

This study identified 158 significantly upregulated DEGs in both LUAD and MI, which suggested that the two diseases may share similar molecular pathological mechanisms. In our results, inflammation-related genes demonstrated a trend of high expression in both diseases, indicating that inflammatory response may play an important role in the occurrence and development of LUAD and MI. Inflammatory responses can affect disease progression by activating multiple signaling pathways that promote cell proliferation, migration, and apoptosis. TNF-α-dependent lung inflammation could promote cell proliferation by upregulating superoxide dismutase-2 (SOD2) expression in LUAD cells [32]. TNF-α-induced inflammation downregulates the expression of CD74 in tumor cells and that of CD74 in macrophages, contributing to the development of LUAD in alveolar II cells [33]. When MI occurs, inflammatory response inhibitory processes in cells are impaired, spatial inhibition of the inflammatory response is disturbed, and TNF-α expression in necrotic cells is uncontrolled [34]. The inflammatory response in patients with MI can be alleviated by TNF-α inhibitors [35]. Functional enrichment analysis demonstrated that these genes were mainly enriched in the IL-17 signaling pathway, which plays an important role in regulating inflammation and immune responses. IL-17 can enhance the inflammatory response by promoting cytokine production, thereby affecting the tumor microenvironment and atherosclerosis formation [36]. Rupture of atherosclerotic plaques within coronary arteries can lead to MI, which is responsible for significant morbidity and mortality worldwide [37]. These results suggested a possible mechanism between the interaction of MI and LUAD through inflammatory responses.

Based on the PPI network, we identified important genes between LUAD and MI, and MMP9 was the only gene that showed a consistent expression trend in multiple datasets and was, therefore, selected as the biomarker for MI and LUAD. MMP9 also showed better disease prediction performance for both diseases. A comprehensive analysis of DNA methylation and gene chip expression data has identified MMP9 as an important pathogenic gene for LUAD [38]. MMP9 is an important factor in LUAD metastasis, with a high MMP9 expression indicating poor survival of LUAD patients. Importantly, MSCs can promote lung cancer cell metastasis by activating the ABL-MMP9 signaling axis [39]. MMP9 has been considered as a biomarker for MI [40]. Cardiac fibroblasts (CFs) are a cell type abundant in the heart and are mainly responsible for cardiac repair function after MI onset. It was found that NKRF could inhibit CF migration and invasion by downregulating the MMP9 level [41]. MMP9 plays an important role in the IL-17 signaling pathway, which is an important pro-inflammatory cytokine that activates multiple inflammatory responses in cells and promotes neutrophil recruitment and activation. MMP9 enhances IL-17-mediated inflammatory responses by catabolizing the extracellular matrix [42]. MMP9 can also promote inflammatory responses by activating the NF-κB pathway. MMP9 is regulated by NF-κB as a downstream effector molecule and enhances NF-κB signaling through a feedback mechanism, thus playing an important role in chronic inflammation and cancer progression [42]. Previous studies reported that elevated activity of MMP9 is associated with atherosclerotic plaque instability, while the rupture of atherosclerotic plaques leads to MI [43]. The high expression of MMP9 in both diseases and its correlation with a variety of inflammatory and immune cells suggested that it can serve as a potential biomarker for the early diagnosis and assessment of disease prognosis. However, future studies could further explore the possible collaboration of neighboring genes in conjunction with the perspective of chromosomal location-function association, providing new insights for a more comprehensive understanding of disease mechanisms.

In addition, we performed molecular docking analysis to identify drugs that may have potential binding effects with MMP9. The binding energies of MMP9 with Ilomastat and Osthole were all greater than 5, indicating a stable binding relationship between them. Ilomastat is an inhibitor of MMP9 [44], and inhibiting the function of MMP9 may reduce the inflammatory response in patients, thereby slowing down the disease process. Osthole suppresses MMP9 expression and controls tumor metastasis by inhibiting the Wnt/β-catenin signaling pathway [45]. This reveals that the binding of Ilomastat and Osthole to MMP9 may trigger a change in its conformation, which affects its active site and substrate-binding ability, providing a new molecular basis for targeting and regulating MMP9 function.

Furthermore, MMP9, a common therapeutic target, has shown the potential to be used to design novel targeted drugs. It has been demonstrated that MMP9 is a common therapeutic target for colitis and colorectal cancer [46]. Based on these findings, we hypothesized that inhibition of MMP9 activity could simultaneously attenuate the inflammatory response and tissue destruction in LUAD and MI, providing a more effective strategy for the treatment of both diseases. Future studies could further explore the protein conformational changes triggered by drug binding using molecular dynamics simulations and verify their specific effects at the molecular and functional levels by gene expression profiling to comprehensively assess the therapeutic efficacy of the drugs and potential side effects.

However, our study has some research limitations. First, the data for this study were mainly obtained from the TCGA and GEO databases, which are public databases with limited sample sizes. Future studies should incorporate larger samples, including clinical data from multiple centers and regions, to improve the generalizability of the findings. Second, the findings of this study are mainly based on bioinformatics analysis, and further *in vivo* or *in vitro* experiments in the future are warranted in order to be able to verify the function and mechanism of MMP9. Finally, this study focused on gene-level analysis and did not address the effects of environmental factors on LUAD and MI and their interactions with MMP9. Therefore, future studies should combine multiomics data as well as environmental exposure data to explore the mechanisms of gene-environment interactions in LUAD and MI to provide a more comprehensive pathological perspective.

## CONCLUSION

Finally, single-cell data analysis further confirmed the expression profile of MMP9 in LUAD, especially its high expression in macrophages. Such a result not only supported the important role of MMP9 in the tumor microenvironment, but also suggested a potential mechanism in tumor immune escape and inflammatory response. Overall, we revealed the common molecular mechanisms of LUAD and MI through bioinformatics analysis and established the importance of MMP9 as a potential common diagnostic marker and therapeutic target. These findings provide a theoretical basis for the development of novel diagnostic and therapeutic strategies for LUAD and MI, but their specific mechanisms and clinical applications still require further validation.

## Figures and Tables

**Table 1:** Molecular docking binding energy.

Term	Spacing	npts	Center
MMP9_Ilomastat	0.675	126, 126, 126	36.885, 38.845, 34.621
MMP9_Osthole	0.675	126, 126, 126	36.885, 38.845, 34.621

## Data Availability

The datasets generated and/or analyzed during the current study are available in the (GSE66360) repository, (https://www.ncbi.nlm.nih.gov/geo/query/acc.cgi?acc=GSE66- 360), (GSE117570) repository, (https://www.ncbi.nlm.nih.gov/geo/query/acc.cgi?acc=GSE117570) and (GSE180678) repository, (https://www.ncbi.nlm.nih.gov/geo/query/acc.cgi? acc=GSE180678).

## LIST OF ABBREVIATIONS

LUAD: Lung Adenocarcinoma
MI: Myocardial Infarction
IL-6: Interleukin-6
GEO: Gene Expression Omnibus
DEGs: Differentially Expressed Genes
PPI: Protein-Protein Interaction
MCC: Maximal Clique Centrality
MNC: Maximum Neighborhood Component
EPC: Edge Percolated Component
LASSO: Least Absolute Shrinkage, and Selection Operator Regression
SVM: Support Vector Machines
DSigDB: Drug Signatures Database
CF: Cardiac Fibroblasts
